# Metformin is a potential therapeutic for COVID-19/LUAD by regulating glucose metabolism

**DOI:** 10.1038/s41598-024-63081-0

**Published:** 2024-05-30

**Authors:** Yongwang Hou, Zhicong Yang, Baoli Xiang, Jiangmin Liu, Lina Geng, Dandan Xu, Minghua Zhan, Yuhuan Xu, Bin Zhang

**Affiliations:** 1 Clinical Laboratory, The First Affiliated Hospital of Hebei North University, Zhangjiakou, 075000 Hebei China; 2 Central Laboratory, The First Affiliated Hospital of Hebei North University, Zhangjiakou, 075000 Hebei China; 3 Respiratory Department, The First Affiliated Hospital of Hebei North University, Zhangjiakou, 075000 Hebei China

**Keywords:** LUAD, COVID-19, Metformin, Glucose metabolism, Bioinformatics, Cancer metabolism, Lung cancer, Tumour biomarkers, Infectious diseases

## Abstract

Lung adenocarcinoma (LUAD) is the most common and aggressive subtype of lung cancer, and coronavirus disease 2019 (COVID-19) has become a serious public health threat worldwide. Patients with LUAD and COVID-19 have a poor prognosis. Therefore, finding medications that can be used to treat COVID-19/LUAD patients is essential. Bioinformatics analysis was used to identify 20 possible metformin target genes for the treatment of COVID-19/LUAD. PTEN and mTOR may serve as hub target genes of metformin. Metformin may be able to cure COVID-19/LUAD comorbidity through energy metabolism, oxidoreductase NADH activity, FoxO signalling pathway, AMPK signalling system, and mTOR signalling pathway, among other pathways, according to the results of bioinformatic research. Metformin has ability to inhibit the proliferation of A549 cells, according to the results of colony formation and proliferation assays. In A549 cells, metformin increased glucose uptake and lactate generation, while decreasing ATP synthesis and the NAD^+^/NADH ratio. In summary, PTEN and mTOR may be potential targets of metformin for the treatment of COVID-19/LUAD. The mechanism by which metformin inhibits lung adenocarcinoma cell proliferation may be related to glucose metabolism regulated by PI3K/AKT signalling and mTOR signalling pathways. Our study provides a new theoretical basis for the treatment of COVID-19/LUAD.

## Introduction

Among malignant tumors, lung cancer is the leading cause of illness and death. The majority (80–85%) of lung cancers are classified as non-small cell lung cancer (NSCLC), which includes subtypes such as lung squamous cell carcinoma (LUSC), lung adenocarcinoma (LUAD), and large-cell carcinoma (LCC)^1^. LUAD, which is known for its high prevalence and aggressive nature, exhibits significant heterogeneity and invasiveness. The absence of early recognizable clinical symptoms and efficient diagnostic techniques often lead to the diagnosis of advanced stage LUAD in a majority of patients^2^, and do not satisfy the requirements for surgical resection, which is a major factor in the unfavorable prognosis of LUAD as a whole. Currently, the introduction of immunotherapy has introduced a new approach to the clinical management of lung cancer. Various immune checkpoint inhibitors (ICIs) have shown promising efficacy in clinical trials^3^. However, some patients do not benefit from immunotherapy^4,5^ or develop resistance to medication^6^. In addition, concerns regarding side effects and adverse reactions persist. Glucose deprivation can also lead to energy stress. Cancer cells die more selectively than normal cells. There is growing evidence that metabolic reprogramming plays an important role in tumor development. This metabolic variability and flexibility enable tumor cells to maintain the reduction–oxidation (redox) balance and generate ATP as an energy source biosynthesis process that is essential for cell survival, growth, and proliferation. Targeting the metabolic differences between tumor and normal cells is a promising novel anticancer strategy^7^. Therefore, it is crucial to investigate the mechanisms underlying LUAD pathogenesis, progression, and metastasis and to identify effective biomarkers and therapeutic targets that can significantly enhance the survival rate of patients with LUAD.

The global outbreak of Coronavirus Disease 2019 (COVID-19), caused by severe acute respiratory syndrome coronavirus 2 (SARS-CoV-2), poses a significant public health threat, with an increasing number of countries affected and millions of people at risk^8^. Recent studies have revealed that individuals with malignancies often experience chronic immunosuppression, rendering them more vulnerable to SARS-CoV-2 infection than healthy individuals. This susceptibility frequently leads to poorer prognoses^9^. Consequently, the COVID-19 pandemic presents substantial challenges for cancer diagnosis, treatment, and prognosis^10^. Peter et al. reported that patients with LUAD diagnosed with COVID-19 exhibited unfavorable prognoses due to frailty^11^. Furthermore, research has indicated that both immunotherapy and chemotherapy can induce chronic immunosuppression in patients with lung cancer, which may be exacerbated by COVID-19 infection^12^. COVID-19 is associated with endothelial dysfunction, inflammation, and hypercoagulability, the co-occurrence of which is associated with an increased rate of serious adverse events, including mortality^13^. Therefore, it is imperative to explore potential drugs that are suitable for treating patients with COVID-19/LUAD.

Metformin is one of the most commonly prescribed medications for type 2 diabetes mellitus. Its well-established therapeutic effect combined with its high safety profile and affordability have positioned metformin as a first-line drug option for the clinical management of type 2 diabetes^14^. Several studies have suggested that metformin improves the prognosis of many cancers and reduces cancer morbidity and mortality, including gastric^15^, liver^16^ and breast cancers^17^. Some studies have shown that metformin reduces mortality in patients infected with SARS-CoV-2 virus^18–20^. In patients hospitalized with COVID‑19, metformin can reduce the markers of inflammation, oxidative stress, and thrombosis^21^. Based on this premise, we hypothesized that metformin could be a potential treatment option for patients with COVID-19 and LUAD. We used network pharmacology and various bioinformatics techniques to explore the therapeutic targets and pharmacological mechanisms of metformin in COVID-19/LUAD. Our aim was to determine the potential therapeutic benefits associated with the use of metformin under these conditions.

## Results

### Identification results of COVID-19-related genes

**Figure 1:**
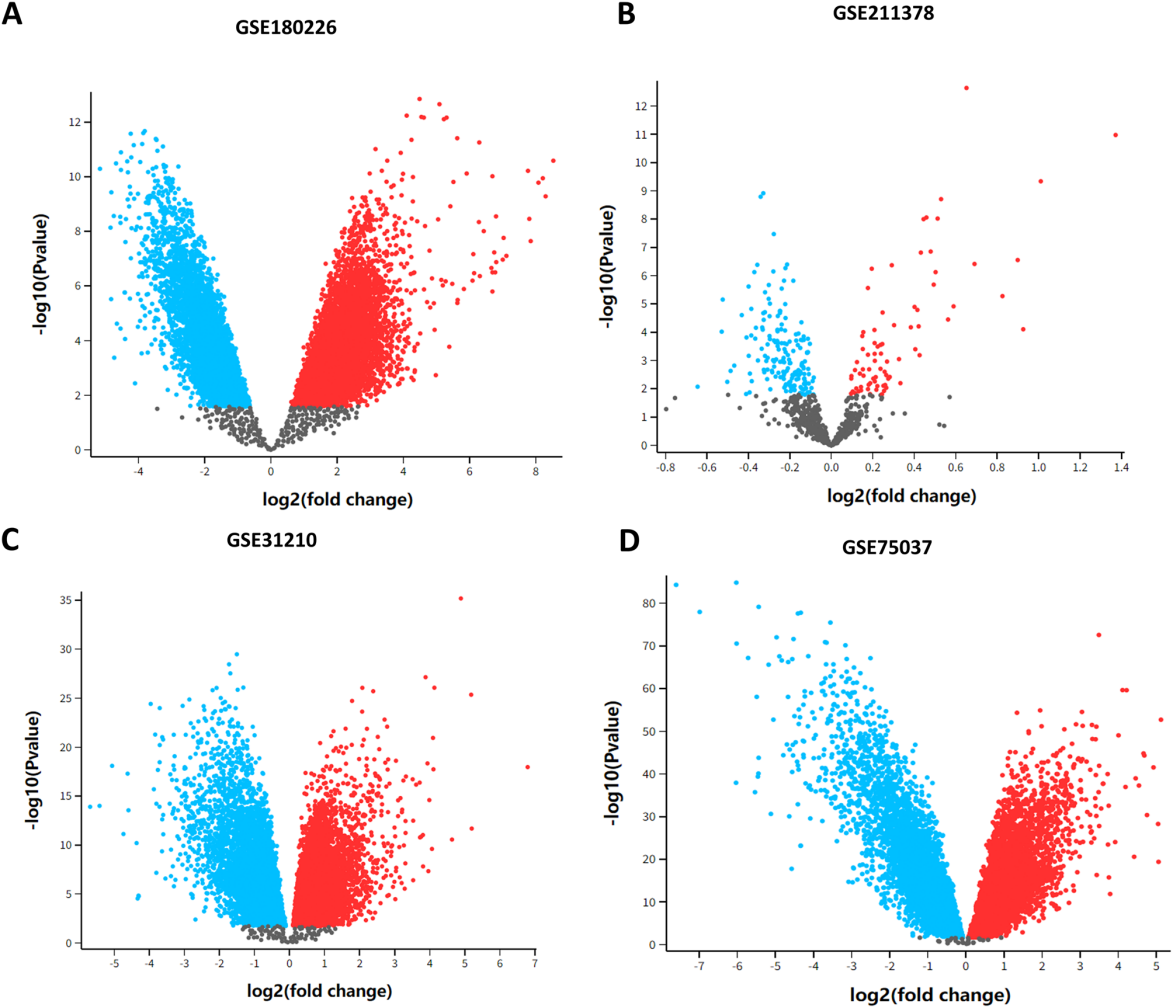
Screening for DEGs from GEO datasets of COVID-19 and LUAD. The red and blue dots represented genes with increased or decreased expression in COVID-19 patients or LUAD patients, respectively, while the black dots represented genes with no significant difference in expression between COVID-19 or LUAD patients. (**A**) Volcano plot of DEGs in the GSE180226 dataset of COVID-19. (**B**) Volcano plot of DEGs in the GSE211378 dataset of COVID-19. (**C**) Volcano plot of DEGs in the GSE31210 dataset of LUAD. (**D**) Volcano plot of DEGs in the GSE75037 dataset of LUAD.

**Figure 2:**
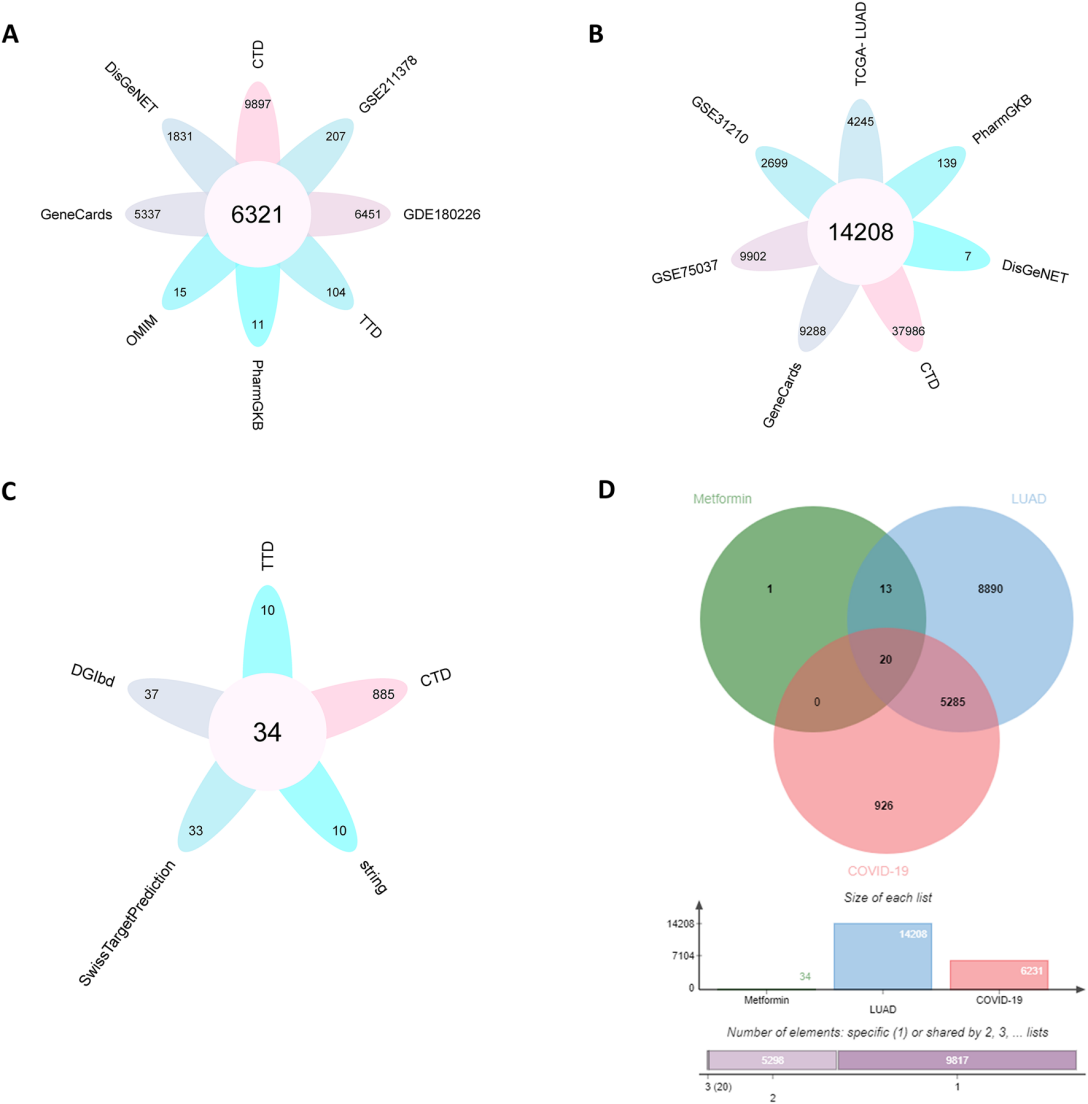
Screening the target genes of metformin for the treatment of COVID-19/LUAD. (**A**) Genes associated with COVID-19 from 2 GEO datasets and six databases. (**B**) Genes associated with LUAD from 2 GEO datasets, TCGA-LUAD dataset and five databases. (**C**) Target genes of metformin from five datasets. (**D**) The target genes of metformin for the treatment of LUAD/COVID-19.

In the GSE180226 dataset, we obtained data from 23 lung tissue samples (containing 20 COVID-19 positive patients and three healthy individuals). In the GSE211378 dataset, we obtained 264 whole-blood samples from 160 COVID-19 convalescent donors and 40 from healthy donors. The DEGs in the GEO dataset were analyzed using GEOR2. 6451 and 207 DEGs, obtained from GSE180226 dataset and GSE211378 dataset (Fig. 1A,B, Supplementary files 1). In addition, 5337, 9897, 104, 15, 1831, and 11 DEGs of COVID-19 were found in GeneCards, CTD, TTD, OMIM, DisGeNET, and pharmaGKB, respectively (Fig. 2A, Supplementary files 1). To ensure the accuracy of the prediction results, genes appearing in two or more databases were classified as those associated with COVID-19. Based on this criterion, 6321 COVID-19-related genes were screened (Fig. 2A, Supplementary files 1).

### Identification results of LUAD-related genes

In the GSE31210 dataset, we included sequencing data from 246 lung tissue samples (226 LUAD tissues and 20 normal lung tissues). In the GSE75037 dataset, we obtained sequencing data from 83 LUAD and 83 matched adjacent nonmalignant lung tissues. A total of 2699 and 9902 DEGs were obtained from the GSE31210 and GSE75037 datasets, respectively (Fig. 1C,D, Supplementary files 2). In addition, 9288, 37,986, 7, 139, and 4245 DEGs of LUAD were found in GeneCards, CTD, DisGeNET, pharmaGKB, and TCGA-LUAD, respectively (Fig. 2B, Supplementary files 2). In this study, we considered DEGs that appeared in at least two databases to be LUAD-related genes, resulting in 14,208 LUAD-related genes. (Fig. 2B, Supplementary files 2).

### Screening the target genes of metformin for the treatment of COVID-19/LUAD

Metformin target genes were explored using five online databases related to drug targets. The results showed that 10, 37, 33, 10, and 885 target genes of metformin were screened from the TTD, DGlbd, Swiss Target Prediction, String, and CTD databases, respectively. To ensure the accuracy of the prediction results, genes appearing in two or more databases were classified as metformin target genes. Based on this criterion, 34 metformin target genes were screened (Fig. 2C, Supplementary files 3). The results of the Venn analysis showed a total of 20 potential target genes of metformin for the treatment of COVID-19/LUAD was obtained (Fig. 2D, Supplementary files 4).

### Gene network analysis and PPI network construction

**Figure 3:**
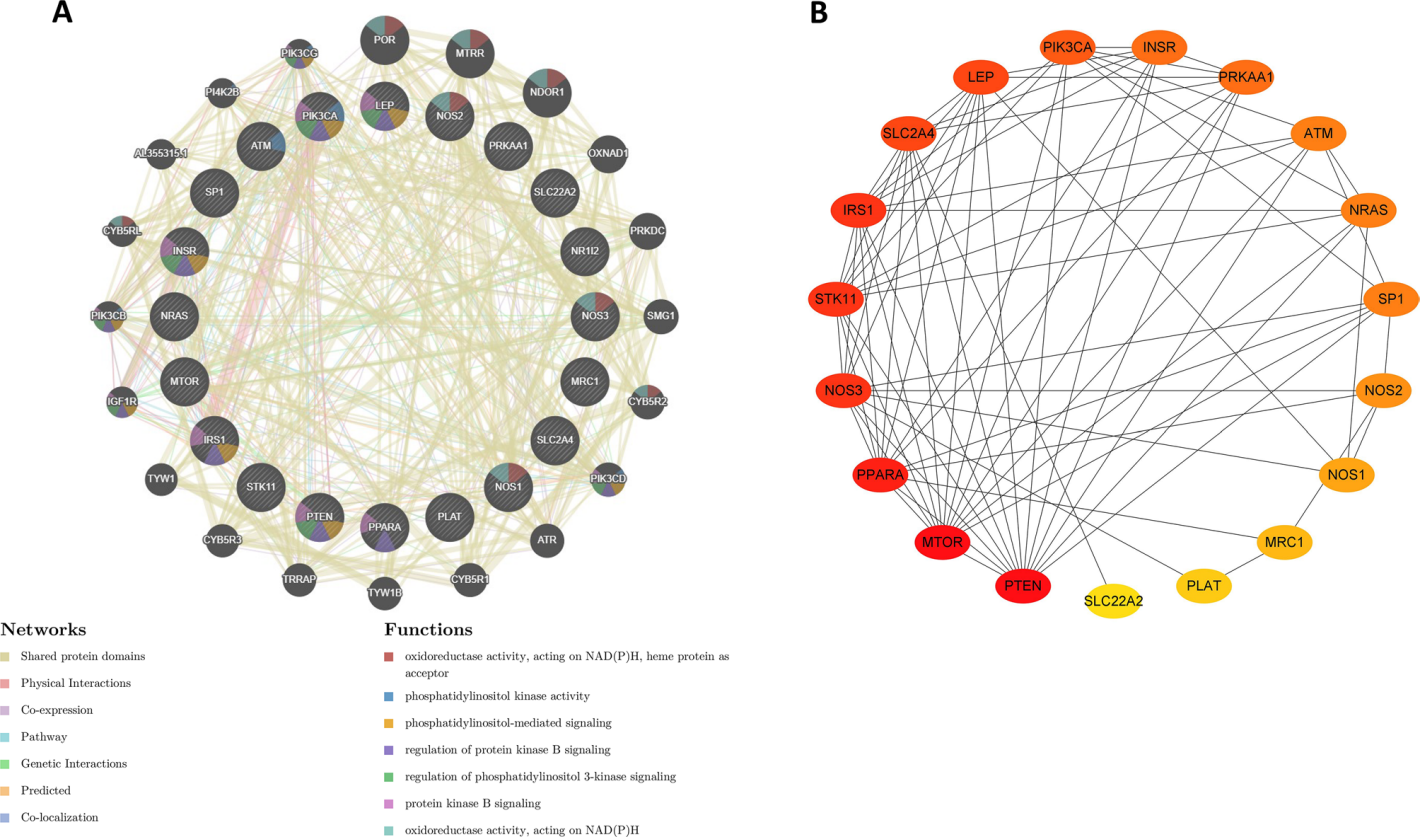
Construction of PPI networks for target genes of metformin for the treatment of COVID-19/LUAD. (**A**) The PPI network was derived from the GeneMANIA database. The different relationships between nodes were indicated by the different colored connecting lines. Nodes were enriched in different functions indicated by the colors of the nodes. (**B**) PPI networks constructed from nodal degree values by Cytoscape software. The degree of the node was proportional to the depth of the node color.

To identify the potential target genes of metformin for the treatment of COVID-19/LUAD, we utilized the GeneMANIA database, which is a comprehensive platform based on multiple transcriptomic and proteomic databases. This platform collects vast amounts of datasets and interactions to discover genes with similar functions and build interaction networks. Our analysis revealed that NADH oxidoreductase activity, phosphatidylinositol-mediated signalling, and protein kinase signalling may be involved in the mechanism of action of metformin in treating COVID-19/LUAD (Fig. 3A). Furthermore, Cytoscape software was used to visualize the degree of each gene in the PPI network, and PTEN, mTOR, and PPARA were identified as core targets for metformin treatment against COVID-19/LUAD due to their high degree within the network (Fig. 3B).

### Gene ontology (GO) and Kyoto encyclopedia of genes and genomes (KEGG) pathway analysis of 20 potential target genes of metformin for the treatment of COVID-19/LUAD

**Figure 4:**
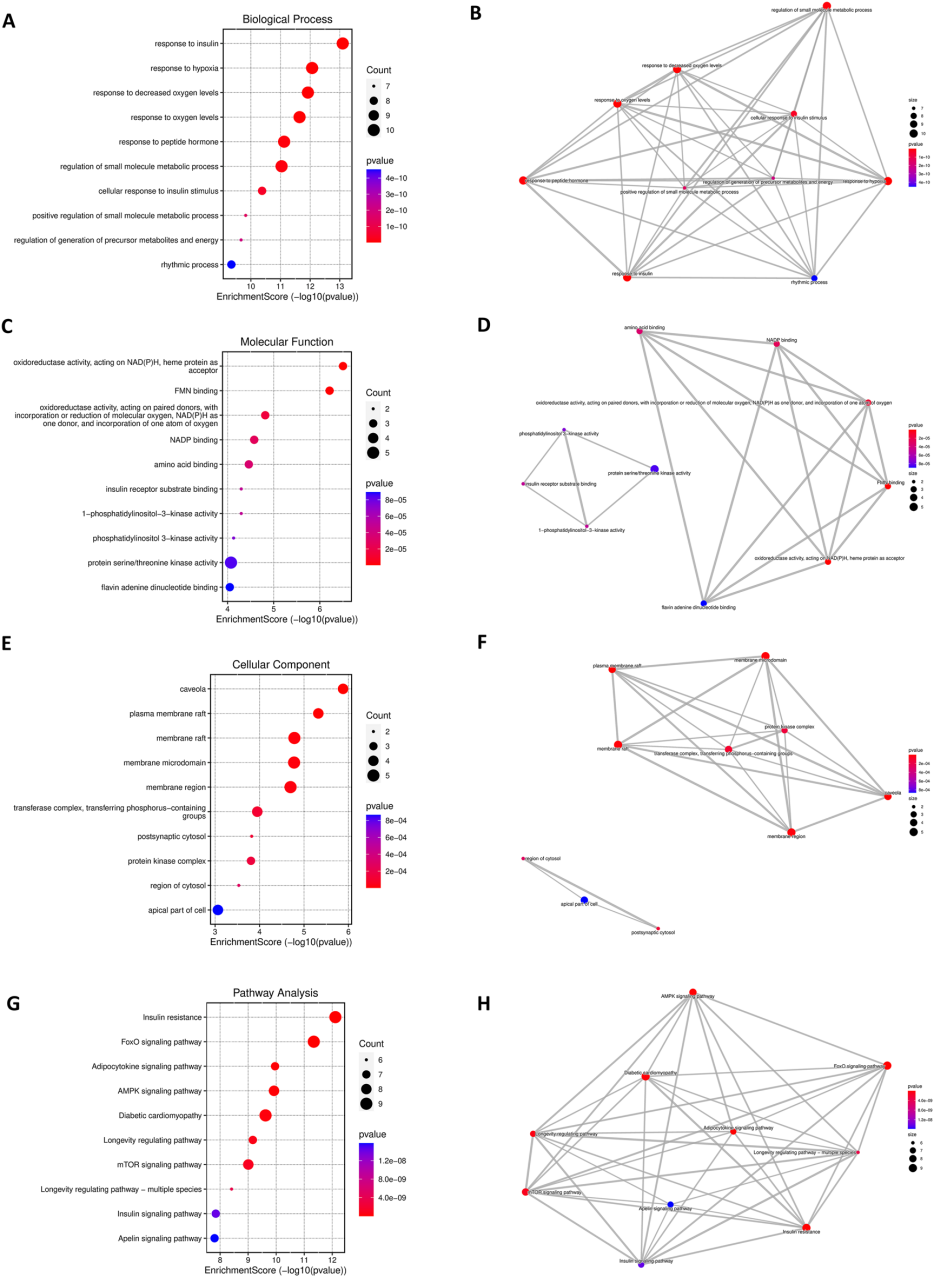
GO and KEGG enrichment analysis of target genes of metformin for the treatment of COVID-19/LUAD. (**A**,**C**,**E**,**G**) The results of BP, CC, MF and KEGG term enrichment analysis, respectively. (**B**,**D**,**F**,**H**) The results of correlation analysis of BP, CC, MF and KEGG term enrichment analysis, respectively.

**Figure 5:**
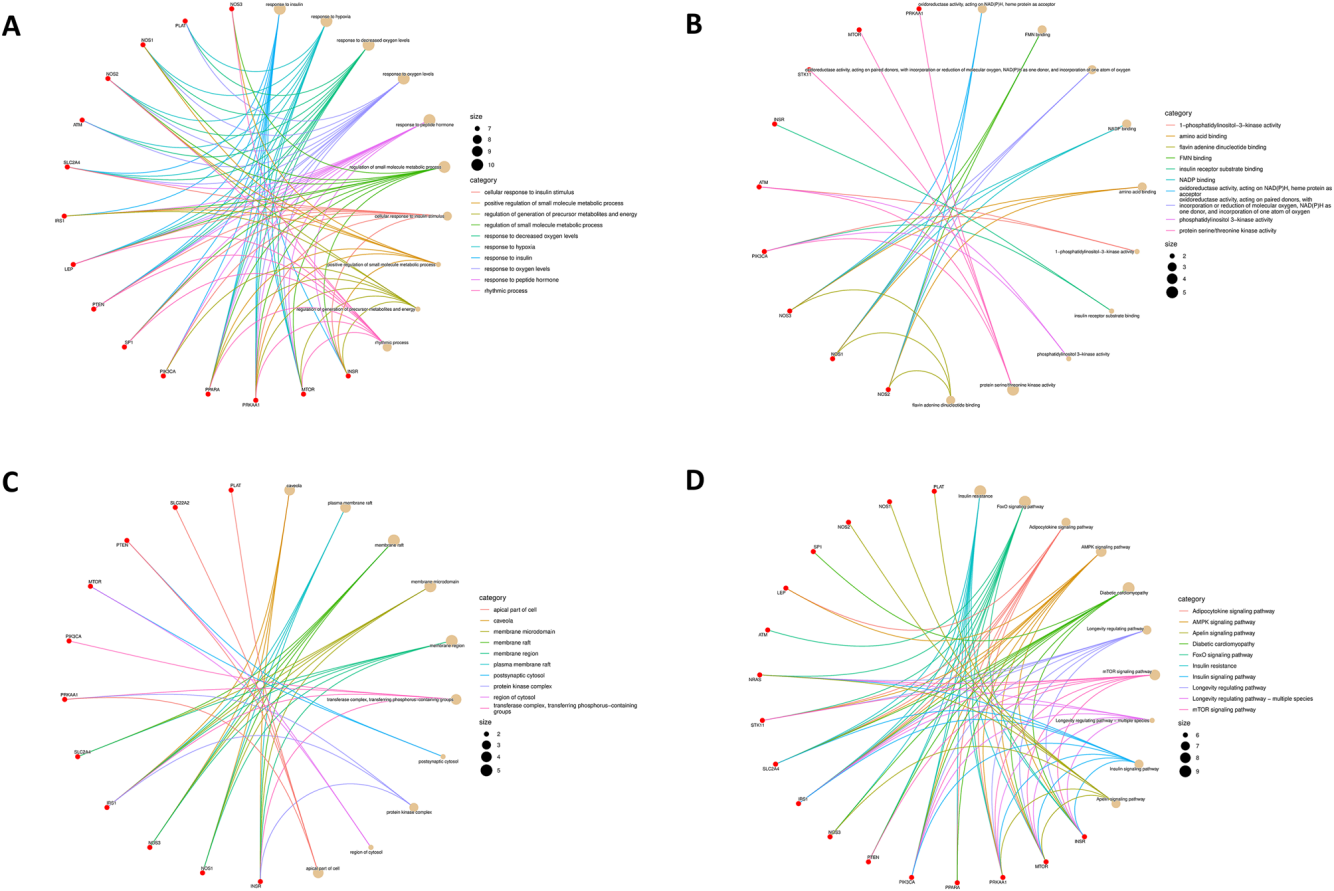
Top ten enrichment analysis results and corresponding genes. (**A**–**D**) The top ten results of BP, CC, MF and KEGG enrichment analysis and corresponding genes.

To investigate the pharmacological mechanism of metformin in the treatment of COVID-19/LUAD, we performed a bioinformatics analysis of 20 potential target genes. The results of GO analysis showed that the BP enrichment results were mainly related to hypoxia, regulation of oxygen levels, and small molecule and energy metabolism (Fig. 4A). The MF enrichment results were mainly related to oxidoreductase NADH activity, phosphatidylinositol activity, and protein kinase activity (Fig. 4C). CC analysis showed that these genes mainly constitute the membrane structure (Fig. 4E). In addition, KEGG enrichment results showed that these genes were mainly involved in the FoxO signalling pathway, AMPK signalling pathway, mTOR signalling pathway and other insulin-related pathways (Fig. 4G). The results of the correlation analysis of BP, CC, MF, and KEGG term enrichment analysis are shown in Fig. 4B,D,F,H. The results of the top ten BP, CC, MF, and KEGG enrichment analyses and the corresponding genes are presented using circus plots (Fig. 5A–D, Supplementary files 5).

### Metformin inhibits the proliferation of LUAD by affecting its energy metabolism

**Figure 6:**
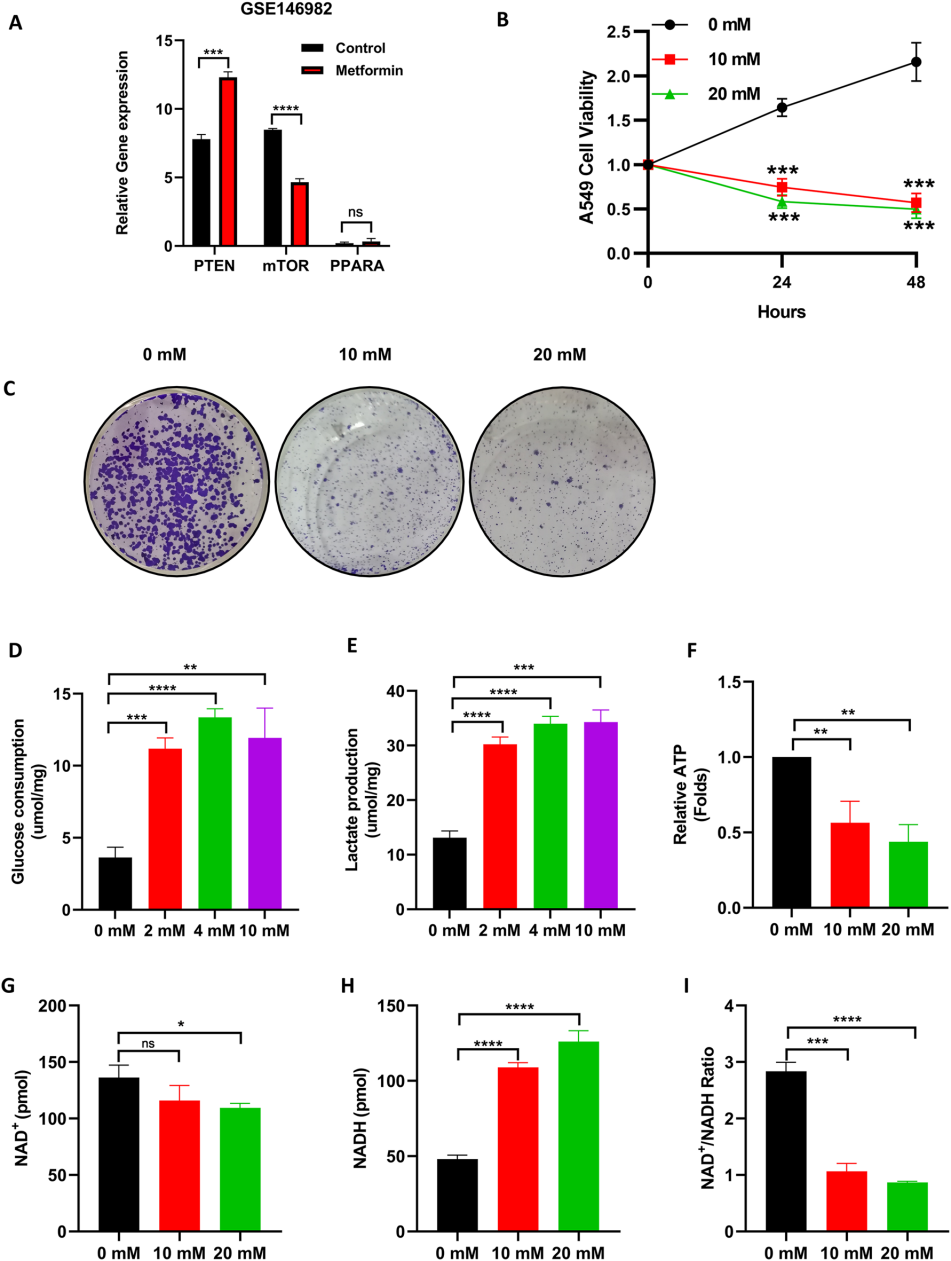
Effect of metformin on energy metabolism in LUAD. (**A**) The expression of PTEN, mTOR and PPARA in A549 cells treated with metformin in GSE146982 datasets. (**B**) Proliferation curves of A549 cells treated with 0 mM, 10 mM, 20 mM metformin, n = 3. (**C**) Clone formation of A549 cells treated with 0 mM, 10 mM, 20 mM metformin. (**D**) Glucose consumption and (**E**) lactate production of A549 cells treated with 0 mM, 2 mM, 4 mM, 10 mM metformin, n = 3. (**F**) ATP, (**G**) NAD^+^, (**H**) NADH and (**I**) NAD^+^/NADH of A549 cells treated with 0 mM, 10 mM, 20 mM metformin, n = 3. *P < 0.05, **P < 0.01, ***P < 0.001, ****P < 0.0001.

First, we examined the effects of metformin on the expression of these three target genes. The results showed that the expression level of PTEN was significantly increased and the expression level of mTOR was significantly decreased in A549 cells treated with metformin, whereas PPARA did not show any significant change (Fig. 6A). Based on the results of the bioinformatic analysis of 20 potential target genes, we suggest that metformin might have effect on energy metabolism in LUAD. The results of the proliferation assay showed that the viability of A549 cells treated with 10 mM and 20 mM metformin was significantly lower than that of the untreated group (Fig. 6B). In addition, the colony formation assay showed similar results, with the number of clones of A549 cells treated with 10 mM and 20 mM metformin being significantly lower than that of the untreated group. (Fig. 6C). To further investigate the mechanism by which metformin inhibited A549 cells, we examined the effects of metformin on glucose consumption and lactate production in A549 cells. As expected, glucose consumption and lactate production of A549 cells were significantly higher compared than in the controls (Fig. 6D,E). Furthermore, in A549 cells treated with 10 mM and 20 mM metformin, ATP production was significantly lower than that in the untreated group (Fig. 6F). As NADH dehydrogenation is an essential part of ATP production, we investigated the effect of metformin on the levels of NAD^+^ and NADH in A549 cells. The results showed that NAD^+^ levels were significantly lower in the metformin group than in the untreated group (Fig. 6G). In addition, NADH in A549 cells treated with 10 mM and 20 mM metformin was higher than that in the untreated group (Fig. 6H). In addition, NAD^+^/NADH levels were significantly lower than those in the untreated group (Fig. 6I). These results indicate that metformin inhibited the production of ATP from the tricarboxylic acid cycle in A549 cells with the aid of depressing NADH dehydrogenation and promoted glycolysis, thereby inhibiting the proliferation of A549 cells.

## Discussion

A growing body of evidence shows that metformin is effective in the treatment and prognosis of lung cancer^22–24^. Some studies have demonstrated that metformin can reduce inflammatory responses in diabetes patients with SARS-CoV-2 infection and has better clinical outcomes^21,25^. Chronically compromised immune systems make patients with LUAD more susceptible to SARS-CoV-2 infection than healthy individuals. Available data confirm that patients with cancer may have a higher risk of SARS-CoV-2 infection and, more importantly, a higher risk of serious complications^26^. Given the antiviral and anticancer properties of metformin, we investigated its target genes and mechanisms in the treatment of COVID-19/LUAD.

We identified PTEN, mTOR, and PPARA as possible core target genes for metformin in the treatment of COVID-19/LUAD by screening genes based on their expression levels. The results of bioinformatics analysis showed that the mechanism of metformin in COVID-19/LUAD might be related to energy metabolism, NADH oxidoreductase activity, FoxO signalling pathway, AMPK signalling pathway, and mTOR signalling pathway. PTEN is a tumor suppressor involved in the regulation of several signalling pathways, including the PI3K/AKT, FoxO, AMPK, and mTOR pathways^27^. Through the regulation of these signalling pathways, PTEN is involved in the regulation of tumor metabolism. Consistent with these results, our findings indicate that by treating A549 cells with metformin, PTEN expression was elevated, while mTOR expression, an oncogene that is frequently upregulated in human cancer^28^, was decreased. In addition, metformin suppressed the proliferation of A549 cells, based on the results of the proliferation and colony formation assays. In a previous study, metformin was found to inhibit the growth of hepatitis C virus-infected cells via mTOR by increasing PTEN and autophagy^29^. These results demonstrated that metformin may inhibit lung adenocarcinoma cell proliferation by altering PTEN and mTOR expression, but we were unable to explore metformin potentially inhibit tumor angiogenesis, metastasis, and chemotherapy resistance. In previous study, Ndembe et al. demonstrated metformin can prevent cisplatin resistance in an inactive liver kinase B1 in vivo model of LUAD^30^. And metformin-repressed NSCLC growth and metastasis by miR-381-YAP-snail axis activity disrupts^31^. Moreover, metformin suppresses tumor angiogenesis and enhances the chemosensitivity of gemcitabine in a genetically engineered mouse model of pancreatic cancer^32^. These studies demonstrated metformin involves multiple signalling pathways and related mechanisms in the treatment of tumors. Because our study was limited to the basic theoretical level and lacked relevant clinical studies, the dosage and side effects of metformin in the treatment of COVID-19/LUAD are unclear. Meanwhile the responsiveness of patients with different stages of LUAD to metformin has not been explored.

As PTEN and mTOR are involved in cellular energy metabolism, combined with the results of our bioinformatics analyses, we conjectured that metformin may exert its cancer-suppressing effects by regulating glucose metabolism. Meanwhile some studies found targeting glucose metabolism could treat COVID-19^33,34^. As expected, metformin promoted glucose consumption and lactate production in A549 cells and decreased ATP production and the NAD^+^/NADH ratio. NAD^+^ is involved in many redox reactions involved in energy generation, glycolysis, tricarboxylic acid (TCA) cycle, oxidative phosphorylation (OXPHOS), fatty acid oxidation, and serine biosynthesis^35^. A higher NAD^+^/NADH ratio is found in cancer cells than in non-cancerous cells, suggesting that NAD^+^ plays an important role in this metabolic conversion^36^. Our results demonstrate that metformin inhibits lung adenocarcinoma cell proliferation by regulating glucose metabolism. Therefore metformin for the treatment of COVID-19/LUAD may work by modulating glucose metabolism. Since PI3K/AKT/mTOR pathway and glucose metabolism are associated with tumor metastasis^37,38^ and tumor angiogenesis^39^, metformin may perhaps be able to treat LUAD by modulating PI3K/AKT/mTOR pathway and glucose metabolism. Further experiments are needed to investigate this.

We defined 20 potential target genes of metformin for the treatment of COVID-19/LUAD as gene set 1 by analyzing data from open databases. Functional analysis of gene set 1 using the GeneMANIA database showed that the mechanism of metformin in the treatment of COVID-19/LUAD may be related to the activity of oxidoreductase NADH, phosphatidylinositol-mediated signalling pathway, and protein kinase B signalling pathway. A growing body of evidence has shown that NAD^+^ mediates both antiviral and anti-inflammatory mechanisms. Increased NAD^+^ levels have been reported to play a role in the prevention and treatment of severe COVID-19 and other viral infections^40^. Moreover, higher NAD^+^/NADH and NADP^+^/NADPH ratios contribute to tumor progression, development, and survival^36^. The phosphatidylinositol-mediated signalling pathway is a key pathway that enhances nutrient uptake, macromolecule synthesis, and cell survival. It is activated in a wide range of cancers^41^. Studies have confirmed that the protein kinase B signalling pathway may promote glycolysis, migration, and invasion of NSCLC cells^42^. In addition, the phosphatidylinositol-mediated signalling pathway and protein kinase B pathway play important roles in the process of viral infection, and the intervention of these signalling pathways contributes to the treatment of viral infection^43–46^. Metformin inhibited the migration and invasion of cancer cells by downregulating the protein kinase B signalling pathway and NAD^+^/NADH ratio^47,48^. These results revealed a potential mechanism of metformin in the treatment of COVID-19/LUAD.

## Conclusions

In this study, we found that PTEN and mTOR could be potential targets for metformin in the treat COVID-19/LUAD. Glucose metabolism regulated by the PI3K/AKT signalling pathway and mTOR signalling pathways may be the mechanisms by which metformin inhibits LUAD cell proliferation. However, our study has several shortcomings and deficiencies. The data used in this study were obtained from a public database, and there is no basic experiment on COVID-19 for verification. In addition, there is a lack of clinical data to support the use of metformin for the treatment of COVID-19/LUAD due to limited funding. Taken together, our study provides a new theoretical basis for treating COVID-19/LUAD.

## Materials and methods

### Flow of research

**Figure 7:**
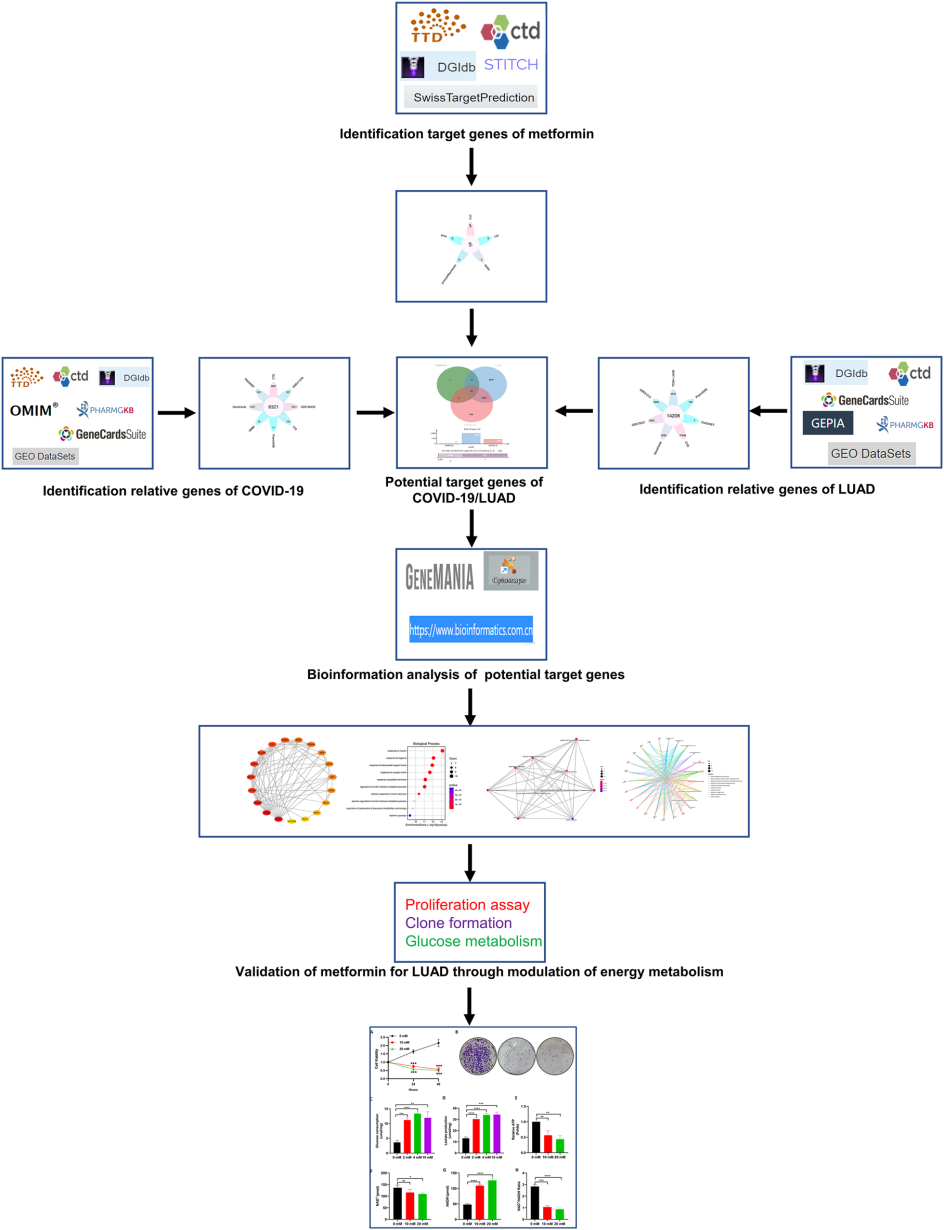
Flowchart. This study follows this flowchart to explore the target genes and molecular mechanisms of metformin for the treatment of COVID-19/LUAD.

As shown in Fig. 7, our study followed a specific flow. First, we conducted a comprehensive search across various databases to identify the genes associated with Metformin, COVID-19, and LUAD. Next, we performed a Venn diagram analysis to determine the potential target genes of metformin for treating COVID-19/LUAD. The identified genes were then subjected to Gene Ontology (GO) enrichment analysis and Kyoto Encyclopedia of Genes and Genomes (KEGG) pathway analysis. Finally, basic experiments were performed to validate the effects of metformin on glucose metabolism in LUAD.

### Screening target genes of metformin

To accurately identify target genes influenced by metformin treatment, we used five reputable online databases related to drug targets: CTD (http://ctdbase.org/), Swiss Target Prediction (http://www.swisstargetprediction.ch/), DGIdb (https://www.dgidb.org/), STITCH (http://stitch.embl.de/), and TTD (http://db.idrblab.net/). By cross-referencing the results of these databases, candidate target genes were selected if they appeared in two or more sources.

### Screening genes of associated with COVID-19

The GSE180226 dataset was obtained from the GEO database specifically related to COVID-19 research, and the differentially expressed genes (DEGs) were analyzed by GEOR2 with filtering criteria (p < 0.01 and absolute value of log fold change > 1). The DEGs of GSE211378 datasets were analyzed using GEOR2 with filtering criteria (p < 0.01 and absolute value of log fold change > 0.1). In addition, the genes associated with COVID-19 were explored using six online databases: GeneCards (https://www.genecards.org), CTD (http://ctdbase.org/), TTD (http://db.idrblab.net/), OMIM (https://omim.org/), DisGeNET (https://www.disgenet.org/), and pharmaGKB (https://www.pharmgkb.org/). To ensure the accuracy of the prediction results, genes appearing in two or more databases were classified as those associated with COVID-19.

### Screening genes of related to LUAD

Two LUAD datasets (GSE31210 and GSE75037) were obtained from the GEO database, and the differentially expressed genes (DEGs) were analyzed using GEOR2 with filtering criteria (p < 0.01 and absolute value of log fold change > 1). Furthermore, five online databases, GeneCards (https://www.genecards.org), CTD (http://ctdbase.org/), TTD (http://db.idrblab.net/), DisGeNET (https://www.disgenet.org/), and pharmaGKB (https://www.pharmgkb.org/) were used to explore the genes associated with LUAD. In addition, the GEPIA (http://gepia.cancer-pku.cn) database was used to explore the DEGs of TCGA-LUAD using filtering criteria (p < 0.01, absolute value of log fold change > 1). To ensure the accuracy of the prediction results, genes appearing in two or more databases were classified as those related to LUAD.

### Construction of PPI network and analysis of hub genes

The GeneMANIA database (http://genemania.org/) was used to construct the protein–protein interaction (PPI) network for metformin target genes in individuals with COVID-19/LUAD comorbidity. To identify the hub genes involved in metformin treatment for COVID-19/LUAD, the Cytoscape software was used to visualize the degree of target genes.

### Gene ontology (GO) term and Kyoto encyclopedia of genes and genomes (KEGG) pathway enrichment analysis

To gain insights into the biological functions and pathways associated with metformin treatment for COVID-19/LUAD, we performed KEGG Pathway Enrichment Analysis and GO analysis, including Biological Process (BP), Cellular Component (CC), and Molecular Function (MF) using the DAVID online analytical tool available at https://david.ncifcrf.gov/summary.jsp. The enrichment analysis results were visualized using online tools provided by http://www.bioinformatics.com.cn.

### Cell culture

The human lung adenocarcinoma cell line A549 was obtained from the American Academy of Sciences and cultured in 1640 medium containing 10% calf serum at 37 °C in an incubator with 5% CO_2_. The medium was changed every alternate day. Experiments were performed when the cells had grown to logarithmic phase.

### Cell proliferation assay

A549 cells (5000 cells/well) treated with metformin (Sigma-Aldrich; Merck KGaA) 0 mM, 10 mM and 20 mM were seeded in 96-well plates and incubated with complete medium for 0 h, 24 h, 48 h. Next, 20 μL of 0.5 mg/mL MTS reagent (Promega) was added to each well on days 0, 1, 2 respectively, and incubate for 1 h. Finally, the absorbance was measured at 490 nm using a μQuant Universal Microplate Spectrophotometer (Bio-Tek Instruments, Winooski, USA). The absorbance was normalized to day 0 data, and proliferation curves were plotted.

### Colony formation assay

Five hundred of A549 cells (500 µL) were plated in triplicate in 6-well plates in RPMI 1640 medium. The medium was replaced every 2 days. Colonies were counted after 6 days.

### Glucose consumption and lactate production

A549 cells were grown to logarithmic phase in 6-well plates and treated with 0 mM, 2 mM, 4 mM and 10 mM metformin for 24 h. The glucose and lactate contents of the fresh medium and the medium after incubating the cells for 24 h were measured using an Automatic Analyzer H7180ID. Glucose consumption and lactate production were then calculated and normalized to the respective cell numbers.

### ATP assay

ATP was measured using the Luminescent ATP Detection Assay Kit (Abcam Co., Ltd.) according to the manufacturer's instructions. Briefly, 1 × 10^6^ cells were resuspended in 100 µL ATP assay buffer. Add 50 µL of the reaction mixture to the standard and sample wells. Then, 50 µL of background reaction mix was added to the sample background control wells. Incubate the plate at RT for 30 min, protected from light. The plate was measured at OD 570 nm for the colorimetric assay and at Ex/Em = 535/587 nm for the fluorometric assay.

### NAD^+^/NADH assay

NAD^+^/NADH ratio was measured using the NAD^+^/NADH Assay Kit (Abcam Co., Ltd.) according to the manufacturer's instructions. Briefly, 2 × 10^6^ cells were homogenized in 200 µL of extraction buffer for NAD^+^ or NADH and neutralized. The concentrations of NAD^+^ and NADH in homogenates were measured using a microplate reader. NAD^+^/NADH levels were normalized to the cell numbers.

### Statistical analysis

Student's *t*-test was used to compare the differences between the two groups, and data are reported as mean values ± SD. The *p*-value < 0.05. Statistical analyses were performed using GraphPad Prism version 9.

### Ethics approval and consent to participate

This study did not require ethical board approval because it did not include human or animal trials.

### Supplementary Information

Supplementary Information 1.

Supplementary Information 2.

Supplementary Information 3.

Supplementary Information 4.

Supplementary Information 5.

Supplementary Legends.

## Data Availability

The datasets presented in this study can be found in the online repositories, including CTD (http://ctdbase.org/), Swiss Target Prediction (http://www.swisstargetprediction.ch/), DGIdb (https://www.dgidb.org/), STITCH (http://stitch.embl.de/), and TTD (http://db.idrblab.net/), GeneCards (https://www.genecards.org), OMIM (https://omim.org/), DisGeNET (https://www.disgenet.org/), GEPIA (http://gepia.cancer-pku.cn), GeneMANIA database (http://genemania.org/) and pharmaGKB (https://www.pharmgkb.org/).

## Abbreviations

LUAD: Lung adenocarcinoma
COVID-19: Coronavirus disease 2019
SARS-CoV-2: Acute Respiratory Syndrome Coronavirus 2
DEGs: Differentially Expressed Genes
GO: Gene Ontology
KEGG: Kyoto Encyclopedia of Genes and Genomes
