# Supplementary material for: Metformin is a potential therapeutic for COVID-19/LUAD by regulating glucose metabolism
Source: Sci Rep. 2024 May 30;14:12406. doi: 10.1038/s41598-024-63081-0 (PMC11137110; doi:10.1038/s41598-024-63081-0)
Supplement: Supplementary file 6 — Supplementary Legends. [file 41598_2024_63081_MOESM6_ESM.docx]

Supplementary files 1. Identification results of COVID-19-related genes.

Supplementary files 2. Identification results of LUAD-related genes.

Supplementary files 3. Screening the target genes of metformin.

Supplementary files 4. Screening of target genes of metformin for the treatment of COVID-19/LUAD.

Supplementary files 5. GO and KEGG analyses of 20 potential target genes of metformin for the treatment of COVID-19/LUAD.
